# Foreign Language Syndrome: Neurological and Psychiatric Aspects

**DOI:** 10.3390/neurolint17080122

**Published:** 2025-08-06

**Authors:** Ansam Eghzawi, Ali Madha, Rany Aburashed

**Affiliations:** 1 Neurogen Biomarking LLC, Dover, DE 19901, USA; rany.aburashed@iinn.com; 2 Insight Hospital and Medical Center, Chicago, IL 60616, USA

**Keywords:** aphasia, foreign language syndrome, speech disorder

## Abstract

Foreign Language Syndrome (FLS) is a rare neuropsychiatric condition characterized by the sudden, involuntary use of a non-native language, with concurrent loss or suppression of the native language. Distinct from Foreign Accent Syndrome (FAS), FLS often arises acutely following anesthesia, brain injury, or psychological stress. Although neuroimaging typically reveals no structural pathology, functional disconnection within bilingual language control systems has been hypothesized. Case reports suggest contributions from both neurological disruptions—such as transient cortical dysfunction—and psychiatric mechanisms, including dissociation and conversion phenomena. This review synthesizes the clinical features, diagnostic strategies, neurocognitive models, and psychiatric interpretations of FLS. It emphasizes the importance of multidisciplinary evaluation and treatment and outlines prognosis patterns. The need for longitudinal follow-up, functional imaging studies, and centralized case databases is highlighted to better understand the pathophysiology and clinical management of this enigmatic syndrome.

## 1. Introduction

Foreign language syndrome (FLS) is defined as the sudden, transient switch from a person’s native language to a second language [1,2,3,4,5,6,7,8,9,10,11]. It is distinct from Foreign Accent Syndrome (FAS), in which patients continue to speak their native tongue but with altered prosody or pronunciation that sounds foreign [12]. In FLS, by contrast, a bilingual or multilingual individual fixes on a non-native language for a period of time. This phenomenon almost invariably occurs in the context of a neurologic insult or perioperative event [1,2,3,4,5,6,7,8,9,10,11].

In addition to its role in pathological phenomena such as Foreign Language Syndrome (FLS), bilingualism has garnered attention for its potential neuroprotective effects across the lifespan. A growing body of evidence suggests that bilingual individuals may experience enhanced cognitive reserve, potentially delaying the onset of age-related neurodegenerative diseases such as Alzheimer’s disease (AD) and other dementias. Studies have demonstrated that bilingualism is associated with later clinical manifestation of dementia symptoms, even in the presence of comparable levels of neuropathology to monolingual individuals [13,14]. Functional neuroimaging also shows that bilinguals may engage broader or more efficient neural networks during cognitive tasks, possibly contributing to resilience against age-related decline [15].

Case reports typically describe FLS arising after general anesthesia or acute brain events [1,2,3,4,5,6,7,8,9,10,11]. In FAS, the underlying speech network (often left hemisphere motor speech areas) is disrupted causing accent change, whereas in FLS the linguistic system accessed shifts entirely to a different language [12]. This review aimed to describe the clinical presentation, neurological aspects, psychiatric aspects, mechanisms and hypotheses, diagnosis and assessment, management and treatment, and future directions and research gaps of FLS.

## 2. Methods

To ensure a comprehensive and representative synthesis of the available literature on Foreign Language Syndrome (FLS), we performed a narrative review using a systematic search strategy across multiple academic databases. Searches were conducted in PubMed, Scopus, and Web of Science from inception through March 2025. The search terms included combinations of “Foreign Language Syndrome”, “bilingualism and neurology”, “language switching”, “bilingual aphasia”, “foreign accent syndrome”, and “conversion disorder and language”. Additional filters were applied to include only peer-reviewed articles in English. Reference lists of relevant articles were also manually screened to identify additional cases and reviews. Although this was not a systematic review per se, we aimed to provide a structured overview of clinical presentations, mechanisms, and management strategies for FLS based on available case reports and theoretical literature.

## 3. Clinical Presentation

Patients with foreign language syndrome (FLS) typically exhibit a sudden, involuntary switch from their native tongue to a secondary language in which they have some proficiency, accompanied by a transient inability to speak or understand their mother tongue [1,2,3,4,5,6,7,8,9,10,11]. Speech during FLS episodes is usually fluent and uses real words from the second language (albeit often imperfectly), not nonsensical syllables [8,11]. For example, Salamah et al. [9] describe a 17-year-old who awoke from surgery unable to use Dutch and for ~24 h could communicate only in English (a language he had learned at school) [9]. Importantly, there is no frank aphasia in the native language—patients generally retain intact grammar and vocabulary for both languages once the episode resolves [8,9]. Some patients are initially confused or disoriented during the episode (akin to emergence delirium), and they may have no awareness of the language switch at the time [9,11].

## 4. Symptom Duration and Variability

Reported FLS episodes have shown marked variability in duration [1,2,3,4,5,6,7,8,9,10,11]. Most perioperative or anesthetic-associated cases resolve spontaneously within minutes to hours [2,3,6,7]. Indeed, a review of eight cases found episode lengths ranging from ~25 min up to 28 h [9]. For example, the 17-year-old in Salamah et al. [9] recovered his native language by 24 h postoperatively [9]. By contrast, FLS arising in other contexts can persist much longer. Beschin et al. [8] describe a patient whose FLS began after brain injury and did not remit over four years of follow-up [8]. Thus, while many FLS episodes are brief and self-limited, chronic courses have been documented, particularly when FLS occurs with underlying neurologic or psychiatric conditions.

## 5. Neuroimaging Findings

Published FLS cases almost uniformly show no focal structural lesion on imaging [1,2,3,4,5,6,7,8,9,10,11]. In reported series, head Computed Topography (CT) and Magnetic resonance Imaging (MRI) were either normal or demonstrated only chronic changes. For example, Gray et al. [16] found unremarkable noncontrast CT and no acute stroke on follow-up MRI in their bilingual aphasia patient [16]. Salamah et al. [9] similarly noted that the postoperative FLS patient had a normal neurological exam and no EEG or neuroimaging was obtained (no abnormalities were evident) [9]. In other words, acute neuroimaging (CT, MRI) is generally done to exclude stroke or hemorrhage and usually yields negative results in FLS cases. There are no reports of Diffusion-weighted Imaging (DWI) abnormalities or perfusion deficits in these patients.

Functional imaging including Functional Magnetic Resonance Imaging (fMRI), Positron Emission Tomography (PET), and Single Photon Emission Computed Tomography (SPECT) during an actual FLS episode has not yet been reported. However, normative studies of bilingual brain function provide indirect insight. In bilinguals, neuroimaging reveals that the same anatomical areas may contain distinct subregions for each language. For example, Kim et al. [17] showed via fMRI that in late bilinguals a second (adult-acquired) language activates a partly non-overlapping portion of Broca’s area compared to the native language, whereas both languages largely co-localize in Wernicke’s region [17]. This suggests that a focal insult to one language-specific network (e.g., the Language 1 representation in Broca’s area) could leave the other network intact and available [17]. As noted above, fMRI of healthy bilinguals shows distinct activation foci for language 1 vs. language 2 within the left frontal lobe.

In a simple word, people who learn a second language later in life, the brain often uses different areas—especially in Broca’s region—for each language. However, when both languages are learned early in childhood, the brain tends to use the same areas for both. This means that the age at which a person learns a second language can affect how the brain stores and processes it. Supporting this, Kuhl et al. [18] found that babies are born able to hear all language sounds, but their brains gradually focus on the sounds they hear the most [18]. This early exposure shapes how their brain develops language pathways. These findings help explain how, in Foreign Language Syndrome, a person might lose access to their native language temporarily while still being able to speak a second one, especially if it is stored in a different brain area.

Functional neuroimaging studies of bilingualism, particularly those using fMRI and EEG, have provided key insights into how the brain manages multiple languages under normal conditions—insights that are crucial for distinguishing typical bilingual activation patterns from the disrupted language access seen in FLS. Recent evidence has shown that bilingualism induces structural brain changes that follow dynamic, non-linear patterns consistent with principles of experience-based neuroplasticity [19]. Specifically, Korenar et al. [19] demonstrated using functional MRI that increased bilingual experience is associated with volumetric changes in subcortical structures involved in language control, such as the caudate nucleus, putamen, and thalamus [19]. These changes follow an expansion–renormalization trajectory: early bilingual exposure leads to structural increases, which then plateau or decrease with continued experience, reflecting more efficient neural processing [19]. This dynamic view helps distinguish normal bilingualism from conditions like FLS, where such adaptive patterns may be disrupted or absent. Incorporating this perspective provides a more current and mechanistic understanding of how the bilingual brain differs from pathological language switching seen in FLS.

Tao et al. [20] demonstrated using functional MRI that bilingualism affects brain function by enhancing domain-general cognitive control systems, particularly those involved in inhibition, attentional switching, and conflict monitoring [20,21,22,23,24,25,26,27,28,29,30]. These systems are supported by the frontal cortex, anterior cingulate cortex, and parietal regions, which are engaged when bilingual individuals manage competing linguistic representations [20,31,32,33,34]. The neural systems for bilingual language control (e.g., suppressing one language while using another) overlap significantly with networks used for general cognitive control [20,35,36,37,38]. This overlap explains why bilingualism may extend benefits beyond language to general cognitive performance [20]. Furthermore, bilinguals tend to show shorter reaction times, greater P3 amplitudes (a marker of attentional resource allocation), and reduced N2 amplitudes (linked to conflict detection) on EEG under certain conditions, which suggests more efficient neural processing during executive control tasks [20,39,40,41,42]. In FLS, patients unexpectedly and involuntarily begin speaking a second language (often one they have not used recently or fluently). This unusual behavior suggests a disruption in the neural mechanisms that normally control language selection and inhibition. From these findings, we can hypothesize that FLS reflects a breakdown in domain-general control networks—specifically, impaired inhibitory control may prevent suppression of the secondary language, dysfunction in attentional switching mechanisms may result in persistent activation of the non-native language, and EEG findings in FLS could show abnormalities in components like P3 or N2, markers of cognitive control, as Tao et al. [20] describe in typical bilinguals. Thus, instead of viewing FLS as merely a linguistic anomaly, it can be framed as a neurocognitive dysregulation involving systems that Tao et al. [20] show are modulated in bilinguals through experience-dependent plasticity. FLS may arise when the neural balance between these control systems is disrupted, such as by stroke, brain injury, or seizure.

Complementary EEG studies using event-related potentials (ERPs) have identified distinct temporal dynamics during language 1 and language 2 processing. For instance, components such as the N400 and P600 vary depending on language proficiency and task type, reflecting semantic and syntactic processing differences across languages [43]. A study by Bermúdez-Margaretto et al. [44] provides neurophysiological (EEG) evidence supporting the hypothesis that FLS may result from abnormal timing, regulation, or coordination within the bilingual language network [44]. In bilingual individuals, both the native (L1) and second (L2) languages are activated simultaneously during word processing [44]. This was demonstrated by modulation of the N400 event-related potential (ERP) component—typically associated with semantic processing—indicating interaction between L1 and L2 meanings. Notably, for the first time in bilinguals, a semantic priming effect was detected as early as 40–60 milliseconds after stimulus presentation, significantly earlier than previously documented. This early effect, observed over centro-posterior scalp regions, reflects rapid and automatic semantic activation across languages. Source reconstruction localized this activity to a left-hemispheric network involving the middle and superior temporal cortices and the angular gyrus, regions known to be central to semantic processing. In addition, conventional N400 effects (~300–450 ms) were observed for both semantic and phonological similarity, reflecting later, controlled stages of lexico-semantic integration. These findings suggest that, in healthy bilinguals, language access is both rapid and parallel, involving a coordinated neural network that supports automatic (early) and controlled (late) stages of processing. In the case of FLS, this balance may be disrupted. For example, damage to the temporal cortex or angular gyrus could impair automatic regulatory mechanisms, resulting in inappropriate activation of one language. Alternatively, dysfunction in frontal or parietal executive regions may compromise top-down control, leading to involuntary language switching. Thus, FLS may reflect a breakdown in the neural systems that typically manage bilingual language regulation.

A recent dual-EEG study investigating Chinese–English bilinguals provided valuable insight into how bilingual language control processes, particularly conflict monitoring and inhibition, dynamically interact with domain-general cognitive control systems [45]. Participants engaged in a task involving both language production and comprehension across three linguistic contexts: single-L1 (Chinese), single-L2 (English), and mixed-language use. Each trial was followed by a flanker task to assess cognitive control. The findings revealed that delta and theta-band synchronization—neural signatures associated with attentional control and cognitive effort—were significantly greater in the L2 and mixed-language contexts compared to the L1-only context. Notably, theta synchronization peaked in the mixed-language condition, reflecting heightened cognitive demand during language switching. Furthermore, participants demonstrated enhanced inhibitory control on the flanker task following L2 and mixed-language trials, suggesting a carry-over effect wherein engaging bilingual language control enhances performance on non-linguistic executive tasks. These results highlight the flexible and context-sensitive nature of bilingual cognitive control systems. In relation to FLS, these findings support the hypothesis that FLS may result from a disruption of this tightly integrated control network. Damage to frontal, cingulate, or fronto-parietal regions involved in theta synchronization and executive regulation could impair inhibition of the non-dominant language, leading to pathological switching and involuntary dominance of the second language. This reinforces the view that FLS is not merely a linguistic disorder, but a manifestation of dysregulated bilingual control mechanisms.

In contrast, patients with FLS exhibit abnormal access to control systems, not due to structural lesions but likely due to transient disruption in language control networks or their regulation (e.g., by frontal-executive systems during emergence delirium or psychiatric states). The absence of functional neuroimaging during FLS episodes remains a critical gap in the literature. Future studies could use bilingual task-based fMRI or EEG during or shortly after FLS episodes to test hypotheses about selective network suppression or executive control failure. Such investigations could clarify whether the brain’s L1-specific circuitry is hypoactive during FLS while L2 circuits remain functional—supporting theories of selective inhibition failure or transient disconnection.

One would predict that during an FLS event the active language network (the emergent language 2) would show normal metabolism, whereas the suppressed language 1 network might appear hypoactive on PET or SPECT. To date, no functional neuroimaging studies in FLS have been published. By contrast, foreign accent syndrome cases often show left fronto-insular hypoperfusion, but accent syndrome is a motor speech disturbance rather than true language switching [46].

## 6. Hypothesized Neural Mechanisms

Understanding the hypothesized neural mechanisms behind FLS requires a foundational appreciation of how bilingualism is normally represented and regulated in the brain. Bilingual language processing involves a dynamic interplay between shared and distinct neural systems, with both languages typically engaging the perisylvian language network—including Broca’s and Wernicke’s areas—as well as domain-general control regions such as the dorsolateral prefrontal cortex, anterior cingulate cortex, and basal ganglia. These control regions are critical for language selection, inhibition of the non-target language, and task switching [47,48]. Neuroimaging studies have shown that late bilinguals may have partially segregated cortical representations for each language, particularly in frontal areas, while early bilinguals tend to show greater overlap [17,49]. The executive control system plays a central role in modulating access to each language depending on context, proficiency, and intention. Disruption in these systems—whether structural, functional, or neurochemical—can impair this finely tuned balance, potentially leading to pathological language switching, as seen in FLS.

Foreign language fixation implicates the classic perisylvian language network [50]. Broca’s area (posterior left inferior frontal gyrus) mediates speech production, and Wernicke’s area (left posterior superior temporal gyrus) mediates comprehension, with the left arcuate fasciculus linking them [50]. The temporal lobes encode lexical and semantic memory for all learned languages, and the inferior parietal lobe (angular/supramarginal gyri) supports multi-modal integration and working memory for language [50].

The neural mechanisms underlying FLS remain speculative, but several models have been proposed. One is selective network lesion: damage (or transient dysfunction) affecting the neural substrate of one language could force reliance on the other. Given Kim et al.’s [17] findings, if a stroke or metabolic insult selectively disrupts the language 1-specific region in Broca’s area, the language 2-specific region (spatially adjacent but separable) may continue to operate, resulting in exclusive use of language 2 [17]. In Gray & Ernst’s model, hypoperfusion of Broca’s area would abolish the motor arm of speech for language 1 while leaving Wernicke’s comprehension intact [16]. This provides a neural basis for bilingual aphasia: if L1 output is cut off, the brain may default to whatever language network can generate speech [16].

Another concept is control/inhibition failure. Bilingual language use normally involves active control by frontal–basal ganglia circuits to suppress the non-target language [49,51]. The basal ganglia, particularly the head of the caudate nucleus, play a central role in this process by inhibiting previously activated or irrelevant language representations. When this system is disrupted—due to injury, dysfunction, or neurochemical imbalance—language control may break down, resulting in involuntary switching or fixation [52]. For example, studies have shown that stimulation of the caudate head can impair this inhibitory function, making it difficult for individuals to manage language selection [53]. In this context, pathological language switching or mixing—as seen in some cases of bilingual aphasia—reflects a failure to suppress competing languages, whereas pathological fixation on one language, as seen in FLS, may reflect abnormal over-inhibition of the native language system [52]. These mechanisms provide a plausible neural explanation for language disinhibition or suppression in FLS.

A diffuse perturbation (e.g., anesthetic, delirium) might transiently impair this executive control, releasing the otherwise inhibited language. Indeed, many FLS cases occur postoperatively during emergence delirium. Salamah et al. [9] note that delirium frequently causes disorganized speech and irrelevant language expression [9]. In their patient, confusion and misperception of environment accompanied the language switch, raising the possibility that FLS is a phenotype of emergence delirium with language fixation [9]. In such a scenario, diffuse anesthesia-induced dysregulation of cortical networks (particularly frontal projections) could nonspecifically disrupt language 1 processing while inadvertently favoring language 2 speech.

**Figure 1:**
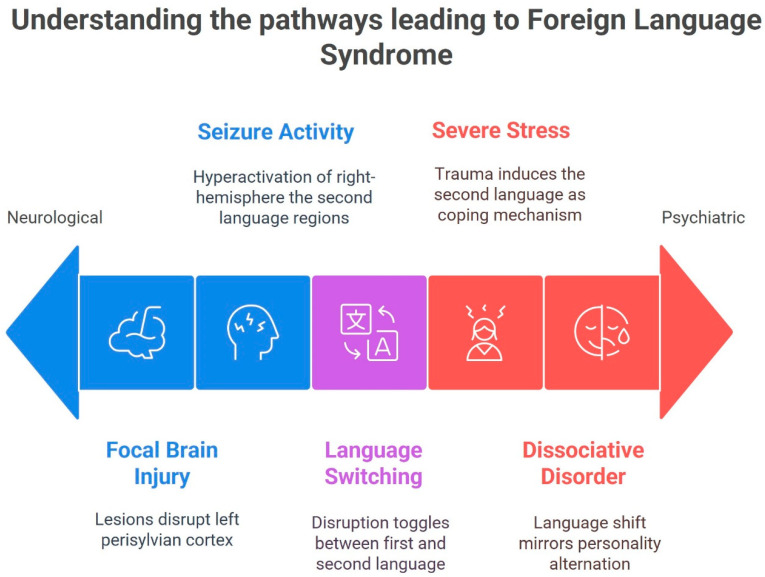
The main pathways leading to Foreign Language Syndrome (FLS).

Memory and encoding factors may also contribute. Early-acquired language 1 and later-acquired language 2 are thought to have different neural representations and emotional context [54,55]. Some have suggested that language 2 (learned academically) may be stored with more rote memorization in distributed cortical areas, whereas language 1 (learned in childhood) might rely on entrenched pathways [54,55]. Thus, a brain insult that preferentially impacts highly practiced networks could paradoxically leave a less-practiced network functional [54,55]. For example, if language 2 representations are more bilateral or right hemisphere-supported in some individuals, then left hemisphere damage might spare language 2. Figure 1 summarizes the main pathways leading to FLS.

## 7. Psychiatric Aspects

FLS often co-occurs with various psychiatric disorders. Case reports and reviews have linked FLS to dissociative/conversion disorders, schizophrenia-spectrum illnesses, and affective (bipolar) disorders. For example, Mendez et al. [56] noted that xenoglossy-like phenomena may appear in dissociative identity disorder (DID) when alternate personalities speak different languages [56]. Likewise, Beschin et al. [8] reported a case of compulsive FLS in a middle-aged man who, after brain injury, compulsively spoke French; this was interpreted as secondary mania (bipolar spectrum) with obsessive–compulsive features [8]. Petrovic et al. describe FLS in a patient with schizoaffective disorder during neuroleptic malignant syndrome, illustrating a link with psychotic mood pathology [11]. More broadly, Mendez et al. [56] highlighted that extreme stress responses (e.g., Ganser syndrome) and conversion reactions to anxiety can produce unusual language symptoms, and that such phenomena are seen in schizophrenia, bipolar disorder, and related conditions [56].

Psychological stressors and trauma appear to precipitate or exacerbate FLS episodes. In most published cases, FLS onset followed acute physiological or psychological insults (surgery, anesthesia, drug reactions) [1,2,3,4,5,6,7,8,9,10,11]. For instance, one 17-year-old developed transient FLS and confusion after orthopedic surgery [9] and a psychotic patient manifested FLS during the stress of neuroleptic malignant syndrome [11]. These perioperative and neuroleptic scenarios suggest that both brain perturbation and emotional arousal play a role. Salamah et al. [9] even proposed that postoperative FLS may belong to the spectrum of emergence delirium, an acute confusional state [9]. More generally, Mendez et al. [56] notes that language distortions (such as the approximate answers in Ganser syndrome) emerge under extreme stress or trauma [56]. By analogy, an individual under severe psychic conflict might unconsciously switch languages as a dissociative or conversion response. Although systematic data are lacking, the temporal clustering of FLS with stressful events supports a psychogenic trigger in many cases. Cases resembling Ganser syndrome, characterized by approximate answers and bizarre language behaviors, often follow major stress events, suggesting that FLS may represent a symbolic or protective escape mechanism from psychological threat [11,56]. Furthermore, the literature on psychogenic aphasia and functional speech disorders provides evidence that language output can be dramatically altered in the absence of organic pathology, particularly in individuals with a history of childhood trauma, PTSD, or dissociative tendencies [57]. These observations support the view that in vulnerable individuals, stress and trauma may temporarily reorganize access to language networks through functional (rather than structural) pathways—resulting in phenomena like FLS.

## 8. Mechanisms of Language Switching

Several psychopathological mechanisms have been proposed that might explain FLS. One involves dissociative or psychotic processes. In schizophrenia or related disorders, patients may encapsulate psychotic content in a second language. Sandoval et al. [58] describe a bilingual schizophrenia patient whose delusional ideas were expressed differently in his native versus second language, suggesting that language context can shape the phenomenology of psychosis [58]. Similarly, dissociative identity disorder can produce xenolalia: Mendez et al. [56] reported cases where alternate identities spontaneously spoke previously learned foreign languages [56]. In these situations, the language shift is coherent within the altered state or delusion (rather than random code-switching).

Other cases point to conversion or compulsion as mechanisms. By this view, FLS is a functional neurological symptom whereby psychological conflict is converted into altered speech output. Mendez noted that conversion reactions to stress (analogous to Ganser syndrome) can yield bizarre language phenomena [56]. For example, Beschin et al’s [8] patient persistently spoke only French after brain injury; this was framed as secondary mania or an OCD-like fixation on being French [8]. In fact, the authors of Mendez’s review explicitly coined the term “compulsive foreign language syndrome” for OCD-driven cases [56]. These observations imply that a pathological compulsion or symbolic “need” to adopt another language (to escape trauma or fulfill a delusion) can drive FLS. In sum, dissociative identity switches, psychotic delusions in a second language, and conversion/compulsive processes have all been invoked as psychogenic mechanisms behind FLS [8,56,58].

## 9. Differential Diagnosis

Foreign Accent Syndrome (FAS): FAS involves a prosodic/accent change in the patient’s native language, not a literal language switch [12,46]. In FAS the patient’s original language is still being spoken (albeit with an altered accent), whereas in FLS the entire spoken language shifts. For instance, Beschin et al. (2016) [8] emphasize that their patient “does not speak Italian with a French accent”—he simply could not speak Italian at all and instead compulsively spoke French [8].

Glossolalia (Speaking in Tongues): Glossolalia (“speaking in tongues”) or spirit possession states can superficially resemble FLS. Glossolalia consists of rhythmic, non-meaningful utterances (often semantically empty) produced in religious contexts [59]. By contrast, FLS output consists of coherent (if error-filled) words from a real language the patient has learned. Beschin et al. [8] note that their patient’s French is maladroit and full of inaccuracies but he does not speak grammelot or gibberish [8], highlighting that FLS speech is structured and meaningful (unlike glossolalic speech).

Bilingual Aphasia: In true aphasia in a multilingual individual, a stroke or lesion causes loss of language ability in one (or both) languages [16]. Classically, bilingual aphasia may selectively impair one language while sparing another [16]. In contrast, FLS is not due to focal brain injury of a language area. The patient’s native language system remains intact (as evidenced by a lack of grammatical errors or dysarthria in that language once recovered). Thus, unlike stroke-related bilingual aphasia, FLS involves a temporary fixation on a non-native language rather than permanent language loss.

Psychogenic (conversion) aphasia: A functional aphasia can produce abnormal speech without neurologic lesion. However, psychogenic aphasia typically yields halting, nonfluent utterances with simplified grammar and preserved comprehension [56]. Patients may give “approximate answers” or speak in a telegraphic style. In contrast, FLS involves fluent, grammatical speech in a different language. The agrammatism of conversion aphasia (broken native speech) differs from the switch to a fully formed second language.

Dissociative identity disorder (DID): In DID, patients may speak a foreign language while others do not [56]. Such xenolalia in DID has been documented. The key distinction is chronic dissociative pathology: multiple identities, amnestic gaps, and other somatoform symptoms accompany the language shift. By contrast, true FLS is transient and typically triggered by a discrete event. Careful history (looking for longstanding dissociation, trauma, childhood onset, etc.) helps distinguish FLS from DID.

## 10. Diagnosis and Assessment

Evaluation of suspected FLS begins with a thorough clinical history and neurological examination. Clinicians should document the time course of language change (often perioperative or acute onset), medications, anesthetic exposures, and any psychosocial stressors. A detailed language history is critical—including native language, second languages, age of acquisition, and proficiency levels. Standardized self-report instruments (e.g., the Language Experience and Proficiency Questionnaire) may be administered to quantify bilingual experience and subjective fluency in each language [60]. Concurrently, cognitive screening with tools like the Mini-Mental State Examination or Montreal Cognitive Assessment helps rule out delirium, dementia, or encephalopathy, and basic neurological testing (orientation, memory, motor/sensory exam) seeks focal deficits [61]. Because many FLS cases arise after anesthesia, neuroimaging (MRI) and EEG are often obtained to exclude acute cerebral insults or seizures; most reported FLS patients have normal MRI and EEG [1,2,3,4,5,6,7,8,9,10,11]. Any abnormalities on initial work-up would prompt a search for stroke, tumor, or toxic-metabolic causes. In asymptomatic or resolving cases, a normal general exam may be the rule, but conservative evaluation is recommended to exclude organic pathologies. Neuropsychological evaluation, speech–language pathology assessment, and psychiatric evaluation may be warranted if the initial evaluation showed any deviation from normal.

Neuropsychological assessment plays a critical role in the comprehensive evaluation of FLS. Beyond basic cognitive screening tools such as the MMSE or MoCA, formal neuropsychological testing allows for detailed profiling of attention, memory, executive function, language fluency, and processing speed. This is particularly relevant in FLS, where subtle cognitive or executive deficits—especially in inhibitory control, working memory, or task switching—may underlie the abnormal language behavior. Such assessments can help differentiate FLS from structural aphasia, psychiatric disorders, or malingering, and may reveal patterns consistent with functional neurological disorders. Furthermore, neuropsychological findings can guide individualized rehabilitation planning and provide objective measures for monitoring recovery over time [47,57]. In cases where language disturbances arise in the context of psychological stress or dissociation, neuropsychological data are also valuable for collaborating with psychiatry and speech–language therapy in a multidisciplinary setting.

## 11. Management and Treatment

FLS requires a collaborative management plan integrating neurology, psychiatry, and speech–language pathology. Neurologists evaluate and address any organic contributors (e.g., stroke or multiple sclerosis) and manage medical precipitants, while psychiatrists/clinical psychologists assess for functional or dissociative factors. Speech–language pathologists (SLPs) perform detailed language assessment and guide communication rehabilitation. In practice, specialized programs for functional neurological disorders routinely employ such teams: for example, an inpatient multidisciplinary clinic tailored each patient’s plan to include cognitive–behavioral therapy (CBT), occupational/physical therapy, neurology and neuropsychiatry input [57]. This integrated model allows treatment to be individualized—for instance, educating the patient about the diagnosis with input from all providers, and then implementing targeted therapies from each specialty (CBT and counseling from psychiatry, language exercises from SLP, etc.) [53,57]. Studies of functional neurological disorders suggest that a coordinated team approach yields better long-term outcomes, and by analogy the same principle applies to FLS [53,57].

No medications have been validated specifically for FLS, so pharmacotherapy is tailored to the underlying cause and comorbid symptoms. Case reports emphasize treating precipitating factors: for example, in one patient who developed FLS during neuroleptic malignant syndrome, full resolution followed cessation of the offending antipsychotic and supportive care with fluids, anticholinergics (biperiden), and lorazepam [11]. Likewise, withdrawal of dopaminergic or anticholinergic agents has been implicated in triggering FLS/FAS, indicating that adjusting or reversing such medications is a key first step [62]. Beyond this, psychiatric medications may be used to control concurrent neuropsychiatric symptoms. All pharmacotherapy should be closely monitored. In general, treatment is symptomatic, and medications are selected based on the overall clinical picture rather than the language deficit alone. Where possible, minimizing polypharmacy and using low doses (titrating slowly) is advised, as patients with functional syndromes can be sensitive to side effects [53]. Importantly, no drug is known to “cure” FLS; medications are used to manage triggers and coexistent psychiatric conditions.

## 12. Prognosis and Recovery Patterns

The clinical course of FLS varies. Published cases suggest that many episodes are acute and self-limiting. In anesthesia-related FLS, recovery is often rapid: for example, a previously healthy teenager who spoke only English for 24 h postoperatively fully regained his native Dutch spontaneously [9]. Similarly, reports of transient post-anesthetic foreign language fixation noted resolution within hours to days without specific intervention [2,3,6,7]. In these acute cases, supportive care and reassurance are the mainstay, and normal speech usually returns.

Overall, available evidence—though limited—indicates that most FLS cases ultimately revert to the patient’s native language. The transient nature of many episodes (especially anesthetic-induced) is encouraging. However, clinicians should remain vigilant for persistent or recurring language changes. In practice, patients are monitored over time, with repeated neurological and psychiatric assessments. Full recovery (remission) is common once precipitating factors are addressed, but even in protracted cases, improvement is expected with consistent multidisciplinary therapy. Further research will be needed to define precise prognostic indicators and optimal therapy duration in FLS.

A summary of all reported cases regarding FLA is provided in Table 1.

## 13. Conclusions

Foreign Language Syndrome occupies a unique intersection between neurology, psychiatry, and cognitive linguistics. Though most cases are transient and benign, its presentation can mimic serious neurological events and warrants careful assessment. Evidence suggests that FLS results from disruption in language control networks—whether through organic lesions, neurochemical imbalance, or psychogenic mechanisms. Clinical management should be multidisciplinary, combining neurological assessment, psychiatric evaluation, and speech–language rehabilitation. While most patients recover their native language spontaneously, persistent or recurrent symptoms may indicate underlying psychiatric or functional pathology. Future research should aim to systematize case reporting, investigate neural correlates via advanced imaging, and explore language-switching mechanisms in bilingual brains. Establishing collaborative registries and conducting longitudinal studies will be critical to advancing diagnostic clarity and treatment strategies for FLS.

## Figures and Tables

**Table 1:** Summary of all reported cases of Foreign Language Syndrome.

Case ID	Age/Sex	Native Language (L1)	Foreign Language (L2)	Comorbidities	Medications	Trigger	Duration	Outcome	Notes	References
Ward & Marshall (1999)	54/M	English	Spanish	None significant	Midazolam, propofol, fentanyl (pre-induction), IV glucose	Post-anesthesia; possible hypoglycemia	Not specified	Full recovery after glucose administration	Repeat episode; L2 learned in school	[2]
Cosgrove et al. (2000)	70/M	English	Hindi	Not reported	Not reported	During induction	Not specified	Full recovery	Denied being able to remember or speak Hindi	[4]
Akpek et al. (2002) Case 1	68/M	Czech	English	Occasional cannabis use	Midazolam, propofol, fentanyl, vecuronium (anesthesia)	Post-anesthesia wake-up test	Not specified	Spontaneous switch back to L1	-	[3]
Akpek et al. (2002) Case 2	-	Turkish	English	Depression, anxiety	Midazolam, propofol, fentanyl, rocuronium	Post-anesthesia	24–28 h	Spontaneous switch back to L1	-	[3]
Webster & Grieve (2005)	55/M	English	Spanish	None reported	Midazolam, propofol, rocuronium, fentanyl, isoflurane	Post-anesthesia for pharyngeal surgery	5–10 min	Spontaneous recovery	Recently returned from Chile	[6]
Ivashkov et al. (2016) Case 1	52/M	English	French	Psoriatic arthritis	Midazolam, fentanyl, propofol, sevoflurane	Post-anesthesia for ankle surgery	~1 h	Full recovery	Partial recall; seizure-like episode observed	[7]
Ivashkov et al. (2016) Case 2	28/M	English	Spanish	Depression, anxiety	Midazolam, fentanyl, propofol, rocuronium, sevoflurane	Post-anesthesia for orbital floor fracture	10 min	Full recovery	Similar episodes previously during alcohol intoxication	[7]
Beschin et al. (2016)	50/M	Italian	French	Hydrocephalus, vascular encephalopathy	Not reported (post-surgical neurologic condition)	Brainstem vascular encephalopathy	Chronic (>4 years)	Persistent compulsive use of French	Secondary mania; compulsive behavior; executive function intact	[8]
Pollard et al. (2017)	64/M	English	Norwegian	Bladder cancer	Propofol, volatile anesthetic, fentanyl	Post-anesthesia for bladder cancer surgery	5 h	Full recovery	Unaware of the language switch	[1]
Salamah et al. (2022)	17/M	Dutch	English	History of fractures, pneumonia, dysphagia; no psych history	Standard anesthesia agents (midazolam, propofol, etc.)—specifics not listed	Post-anesthesia from orthopedic surgery	24 h	Spontaneous recovery	Believed he was in Utah; no memory impairment on follow-up (brief episode of confusion and disorientation)	[9]
Petrovic et al. (2024)	34/M	Serbian	English	Schizoaffective disorder; recurrent psych hospitalizations	Chlorpromazine (trigger for NMS), biperiden, lorazepam	Chlorpromazine-induced NMS	5 min	Full recovery post-NMS treatment	Limited English exposure, never used practically	[11]
Mathis et al. (2024)	21/F	English	Not specified (non-native)	Healthy, no comorbidities	Midazolam, fentanyl, propofol (IV anesthesia)	General anesthesia for wisdom tooth extraction	~24 h	Spontaneous resolution; no intervention required	First female case; occurred with IV sedation agents; no stroke workup needed	[10]
